# Einsatz von Simulationspersonen zur Prüfung von Handlungskompetenz im Bereich der Kinderschutzmedizin im Dritten Abschnitt der Ärztlichen Prüfung

**DOI:** 10.1007/s00112-022-01674-7

**Published:** 2022-12-28

**Authors:** Jonas Steglich, Linn Hempel, Susanna Jaspers, Dietrich Stoevesandt, Marko Weber, Christian Kunze, Susann Weihrauch-Blüher, Caspar Kühnöl, Lina Woydt

**Affiliations:** 1 Dorothea Erxleben Lernzentrum, Medizinische Fakultät, Martin-Luther-Universität Halle-Wittenberg, Magdeburger Str. 12, 06112 Halle (Saale), Deutschland; 2 Institut für Rechtsmedizin, Medizinische Fakultät, Martin-Luther-Universität Halle-Wittenberg, Halle (Saale), Deutschland; 3 Universitätsklinik und Poliklinik für Radiologie, Universitätsklinikum Halle, Halle (Saale), Deutschland; 4 Universitätsklinik und Poliklinik für Pädiatrie I, Universitätsklinikum Halle, Halle (Saale), Deutschland

**Keywords:** Staatsexamen, Simulation, Nationaler Kompetenzbasierter Lernzielkatalog, Kinderschutzmedizin, Kindesmisshandlung und -vernachlässigung, State examination, Simulation, National learning objectives catalogue, Child protection, Child abuse and neglect

## Abstract

In der COVID-19-Pandemie war die Durchführung des Dritten Abschnitts der Ärztlichen Prüfung mit Simulationspersonen möglich. Simulationen ermöglichen die standardisierte Prüfung medizinischer Handlungskompetenz entsprechend den Lernzielen des Nationalen Kompetenzbasierten Lernzielkatalogs. Die Prüfung von Szenarien der Kinderschutzmedizin erscheint zweckmäßig, um die Aufmerksamkeit angehender Ärztinnen und Ärzte für Verdachtsmomente möglicher Kindesmisshandlung zu schärfen. Der vorliegende Fall berichtet über die Simulation eines nichtakzidentellen Traumas im Kindesalter.

## Abstract (en)

During the COVID-19 pandemic, the third section of the medical examination could be performed with simulation patients. Simulations enable standardized examinations of medical competency according to the educational objectives of the national learning objectives catalogue. The evaluation of student’s medical response to child abuse and neglect seems to be an appropriate opportunity to increase attention of prospective physicians for potential child abuse. The presented case reports the simulation of a pediatric nonaccidental trauma.

## Einleitung

Simulationspersonen (SP) sind medizinische Laien, die nach einer entsprechenden Schulung und anhand eines vorgegebenen Rollenskripts einen Patienten für Ausbildungszwecke simulieren können [3]. Der Einsatz im Rahmen der Ausbildung von Medizinstudierenden ist seit mehreren Jahren auch im deutschsprachigen Raum etabliert [7].

Speziell im Kontext der Kinderschutzmedizin kommt der Fallsimulation mittels SP, die hier die Rolle von Angehörigen übernehmen, eine besondere Bedeutung zu, da die Ausbildung Studierender aufgrund medikolegaler Faktoren anhand realer Patienten nicht umsetzbar ist. Um dennoch die Aufmerksamkeit angehender Ärztinnen und Ärzte für Verdachtsmomente möglicher Kindesmisshandlung zu schärfen, erscheint die Konstruktion von Szenarien der Kinderschutzmedizin sowohl im Lernprozess als auch in Prüfungen zweckmäßig.

Die studentische Ausbildung an der Martin-Luther-Universität Halle-Wittenberg (MLU) wird in enger Kooperation zwischen den Kliniken für Pädiatrie und dem Institut für Rechtsmedizin durchgeführt und umfasst semester- und fachübergreifend eine Vorlesung und 2 Seminare mit den Schwerpunkten Kindesmisshandlung und -vernachlässigung. Es werden verdachtssuspekte Befunde und Krankheitsbilder, weiterführende Diagnostik und Aspekte der interdisziplinären Patientenbetreuung sowie mit bei entsprechendem Verdacht einhergehende rechtliche Aspekte gelehrt.

Während der COVID-19-Pandemie war es abweichend von der Approbationsordnung für Ärzte (ÄApprO) [1] regelhaft möglich, den Dritten Abschnitt der Ärztlichen Prüfung (M3) mittels SP durchzuführen [2].

An der MLU wurden die M3-Prüfungen vom Frühjahr 2020 bis zum Frühjahr 2022 anhand von Simulationen durchgeführt. Die Prüfungen sind standardisiert aufgebaut: Zunächst erfolgt ein 30-minütiges (Fremd‑)Anamnese-Gespräch mit einer SP, das per Videoübertragung von den Prüfern verfolgt werden kann. Auf die Anamnese aufbauend ist vom Prüfling innerhalb von 10 min eine diagnostische Strategie zur weiteren Abklärung des Konsultationsanlasses zu entwickeln. Anhand einer vorbereiteten Akte mit weiterführenden Informationen zum Patienten wird schließlich innerhalb von 45 min eine Epikrise verfasst, die der Prüfungskommission vor Beginn des nachfolgenden Prüfungsgesprächs vorgelegt wird und als Basis für das mündliche Prüfungsgespräch dient.

Anhand des nachfolgend geschilderten, auf einer realen Untersuchung basierenden, pädiatrischen Prüfungsfalls wird die Möglichkeit erörtert, wie durch den Einsatz einer SP Handlungskompetenz im Bereich der Kinderschutzmedizin in einer M3-Prüfung geprüft werden kann.

## Falldarstellung

### Simulationsszenario

Die Simulation beginnt in einer Ambulanzsituation. Die SP simuliert eine 26 Jahre alte Sachbearbeiterin, die ihren 12 Monate alten Sohn mit großer Sorge nach einem Vorfall in der Kindertagesstätte vorstellt. Der Prüfling übernimmt die Rolle der aufnehmenden Ärztin bzw. des aufnehmenden Arztes.

### Gesprächsanlass

Die Mutter berichtet, ihr Sohn sei am Vortag beim Spielen im Sandkasten von einem älteren Kind geschubst und mit einer Schaufel auf den rechten Arm geschlagen worden. Seitdem bewege er den rechten Arm vermindert, halte ihn in Schonhaltung und sei allgemein unruhig. Sie äußert im Gespräch den Wunsch nach dem Ausschluss ernsthafter Verletzungen. Sonst habe er „nur ein paar blaue Flecke“ davongetragen. Die Mutter erklärt die Hämatome mit einer möglichen „Bluterkrankheit“ des Großvaters mütterlicherseits.

**Figure 1:**
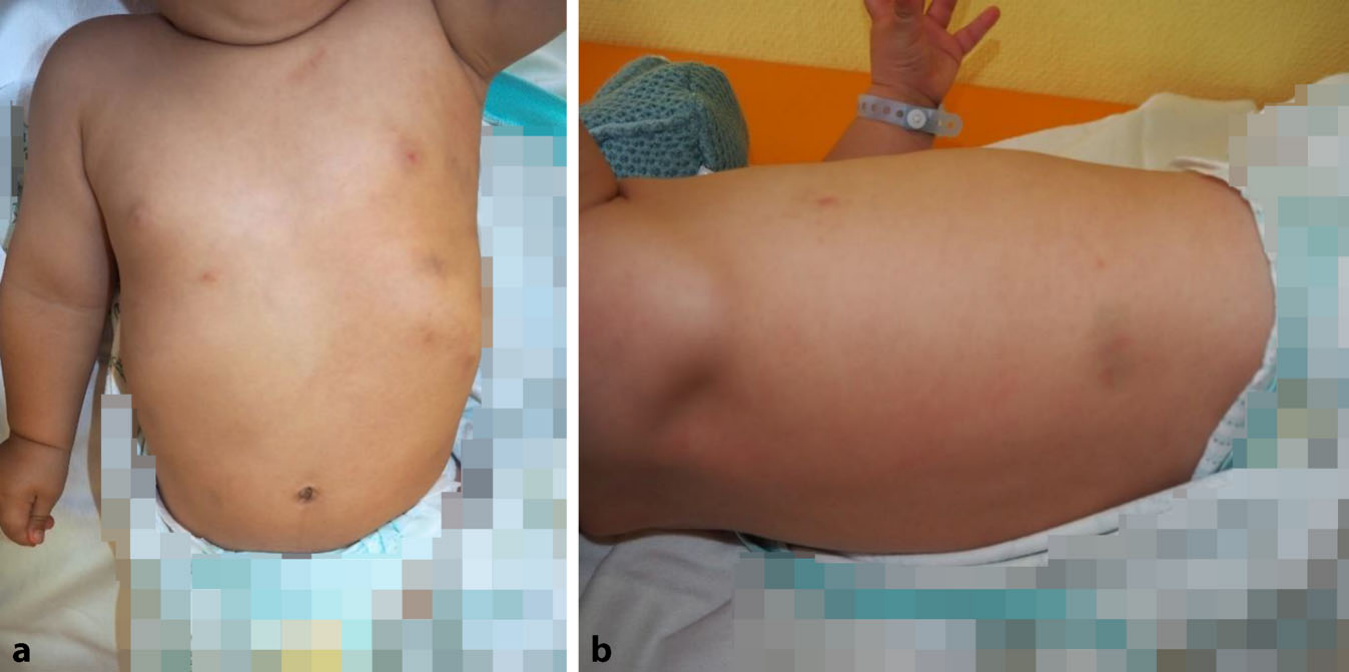

Fotos, die die Mutter am Unfalltag aufgenommen hat, erhält der Prüfling während des Gesprächs (Abb. 1).

### Fallrelevante Angaben zum Patienten

Die Angaben der Mutter zur vegetativen Anamnese sind altersentsprechend unauffällig. Vorerkrankungen sind nicht bekannt. Eine Hämophiliediagnostik ist bisher trotz der auffälligen Familienanamnese nicht erfolgt. Die U12 hat vor 2 Wochen stattgefunden, das Kinderuntersuchungsheft wurde jedoch zu Hause vergessen, sodass sich der Prüfling auf die Angabe der Mutter (SP) verlassen muss, dass der Kinderarzt keine Auffälligkeiten feststellen konnte.

Auf Nachfrage wird von der SP berichtet, dass ihr Sohn und ihr neuer Partner eine Weile gebraucht haben, um sich aneinander zu gewöhnen. Der Partner sei eher distanziert und das Kind weniger lebhaft in seiner Gegenwart. Sie wünsche sich jedoch sehr, dass sich die beiden aneinander gewöhnen, und lässt sie daher regelmäßig zusammen allein. Im Verlauf des Gesprächs macht die SP weitere Angaben zu ihrem Partner, der nicht der Vater des Kindes ist und nicht viel über seine eigene Kindheit spricht. Seine Eltern waren alkoholabhängig, sodass er während seines 14. Lebensjahres ausgezogen ist. Die mangelnde Fürsorge seiner Eltern belaste ihn noch heute.

### Anweisungen für die SP

Die Mutter wird als fürsorgliche Person, der die Gesundheit ihres Sohnes sehr am Herzen liegt, dargestellt. Sie vermittelt den Eindruck eines aufgeschlossenen Elternteils und ist seit dem Vorfall in ehrlicher Besorgnis um das Kind. Sie möchte alle an sie gestellten Erwartungen erfüllen und hat das Ziel, eine intakte Familie zu haben. Sie vertraut Ärzten ganz grundsätzlich und folgt stoisch deren Rat.

### Untersuchungsbefund (Auszug)

12 Monate altes Kleinkind. Gewicht 10 kg, Körperlänge 77 cm. Das Kind wirkt schreckhaft und berührungsempfindlich. Mehrere, über den Rumpf verteilte Hämatome. Druckschmerz über dem mittleren Drittel des rechten Unterarms, jedoch ohne begleitende Weichteilschwellung, kein erkennbares Hämatom.

Der Untersuchungsbefund wurde dem Prüfling schriftlich vorgegeben und stand diesem bereits zum Zeitpunkt der Anamnese zur Verfügung.

### Weiterführende Informationen aus der Patientenakte

**Figure 2:**
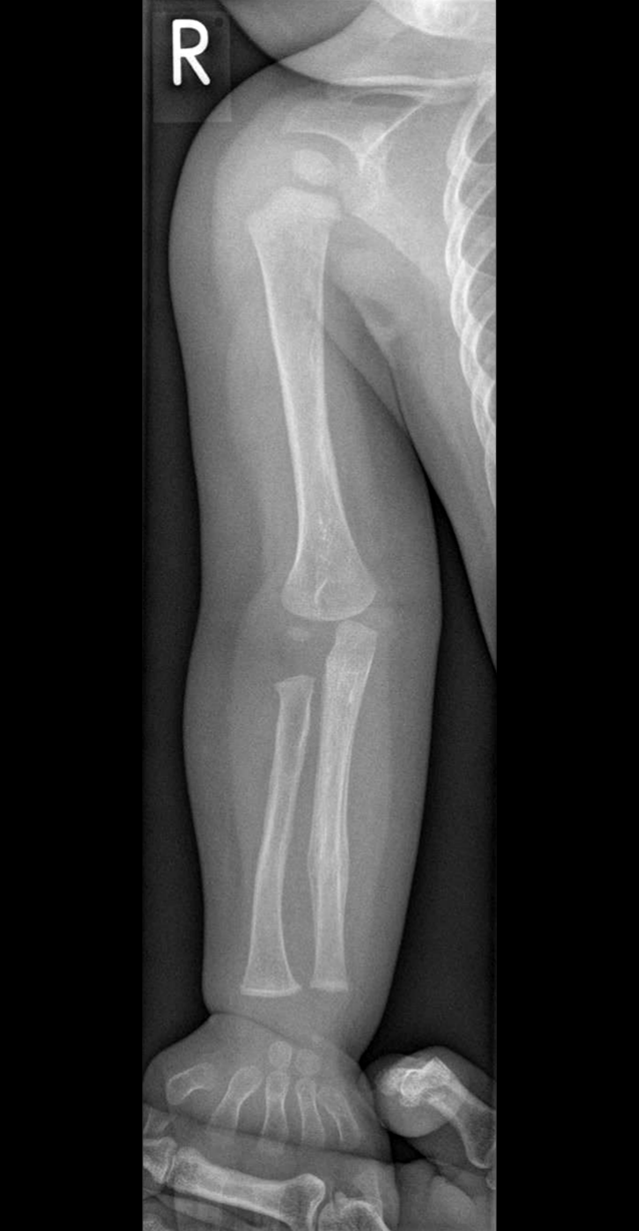

In der Akte befinden sich Perzentilenkurven, mit denen sich die Angaben zu Körpergröße und -gewicht alters- und geschlechtsstandardisiert einordnen lassen. Die Laborparameter schließen eine Hämophilie sicher aus. Weiterhin enthält die Akte das vom Prüfling anzufordernde Röntgenbild des rechten Arms (Abb. 2), inklusive Befund, die eine Fraktur durch das aktuelle Trauma ausschließen, jedoch eine nichtakzidentelle Genese der alten Unterarmfraktur insbesondere unter Berücksichtigung des Alters des Kindes nahelegen.

### Erwartungsbild

Der Prüfling soll unter Zuhilfenahme der von der SP vorgezeigten Fotos das Verdachtsmoment einer Kindesmisshandlung durch stumpfe Gewaltanwendungen erkennen, da der körperliche Untersuchungsbefund durch die anamnestischen Angaben nicht vollumfänglich erklärt werden kann. Gleichzeitig wird erwartet, dass der Prüfling trotz des evtl. erfundenen Behandlungsanlasses eine Röntgenuntersuchung des rechten Unterarms zum Frakturausschluss anordnet. Zusätzlich sollen die familiäre Vorbelastung des Jungen in Bezug auf eine Hämophilie anhand der Familienanamnese erkannt und eine entsprechende Diagnostik eingeleitet werden.

## Diskussion

Im oben beschriebenen Szenario sollen die Verdachtsdiagnose einer nichtakzidentellen Genese gestellt und weitere diagnostische Maßnahmen zur Abklärung des Sachverhaltes eingeleitet werden.

Die vorgestellte Form einer simulationsbasierten M3-Prüfung mit SP ermöglicht die standardisierte Prüfung der Lernziele V.01.1.1.75 und VIII.6-04.4.5 gemäß dem Nationalen Kompetenzbasierten Lernzielkatalog Medizin [5], ohne dabei Rücksicht auf ethische, organisatorische oder medikolegale Einschränkungen eines realen Falles nehmen zu müssen. Im Wesentlichen fordern diese Lernziele, dass eine Absolventin bzw. ein Absolvent am Ende des Studiums die Kompetenz erworben hat, im Falle einer vorliegenden Kindesmisshandlung diesen Konsultationsanlass als solchen zu erkennen, entsprechende Notfallmaßnahmen und weitere Diagnostik einzuleiten sowie (grund-)rechtliche Bezüge zum Kinderschutz benennen zu können und ihr/sein Handeln danach auszurichten.

**Figure 3:**
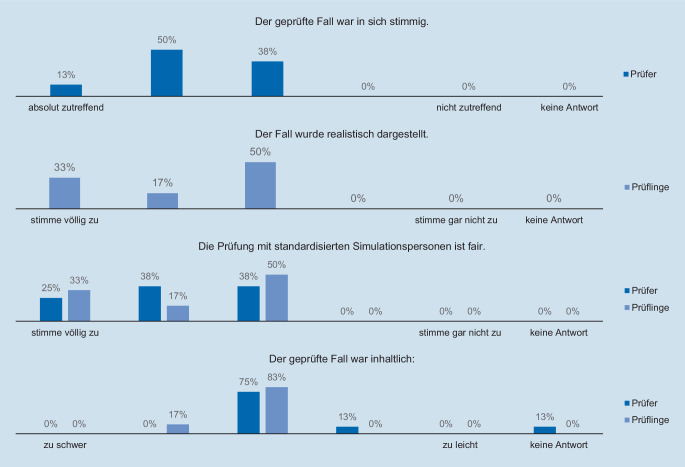

Angelehnt an bisherige Untersuchungen [4] wurden der Fall und die Simulation als Prüfungsformat nach Ende des Prüfungsgesprächs von 2 Prüfungsgruppen, bestehend aus 6 Prüflingen und 8 Prüfern, mittels Fragebogen auf einer 5‑stufigen Likert-Skala evaluiert (Abb. 3).

Die Prüfer beurteilten das Szenario als plausibel und die Prüflinge als realistisch. Die Prüfung mittels SP halten beide Parteien für fair und schätzten den Fall hinsichtlich des Anforderungsniveaus als geeignet ein. Grundsätzlich bietet die Simulation mit Videoübertragung die Möglichkeit, die Interaktion des Prüflings mit der SP standardisiert zu bewerten. In der Beurteilung der Prüfungsleistung wurde sie jedoch nicht berücksichtigt.

Grundsätzlich sind Beschreibungen zu weiteren Simulationen aus dem Bereich der Kinderschutzmedizin bisher selten und eher im englischsprachigen Raum zu finden [6]. Die Durchführung reproduzierbarer, realitätsnaher und hinsichtlich der zu bewertenden Prüfungsleistung vergleichbarer Szenarien erscheint jedoch in Vorbereitung auf die geplanten Änderungen der ÄApprO angemessen. Des Weiteren könnte die wiederholte Anwendung im Lernprozess und in Prüfungen die Aufmerksamkeit für das Vorliegen möglicher Kindesmisshandlungen im ärztlichen Berufsleben erhöhen. Kritisch kann angemerkt werden, dass im Bereich der Pädiatrie Simulationen mit SP auf die Durchführung einer Fremdanamnese festgelegt sind, da Kinder als SP aus rechtlichen Gründen nur schwer einsetzbar sind und Simulatoren modellbedingt mit entsprechenden Einschränkungen einhergehen. In einem SP-Rollentraining vorab wurde das Szenario mehrfach mit und ohne Kleinkindsimulator (auf der Krankenliege liegend) getestet. Die Durchläufe mit Simulator zeigten einen Fokus auf die Symptome und den Simulator, was eine Verunsicherung des Testprüflings sowie ein Improvisieren zur Folge hatte und weniger die Fremdanamnese in den Mittelpunkt nahm. Somit wurde beschlossen, das Szenario zur Entlastung für die Prüflinge ohne den Patienten stattfinden zu lassen.

## Fazit für die Praxis

- Simulationen mit Simulationspersonen ermöglichen die Überprüfung von Lernzielen der Kinderschutzmedizin im Dritten Abschnitt der Ärztlichen Prüfung, ohne dabei Rücksicht auf medikolegale Einschränkungen eines realen Falls nehmen zu müssen.
- Simulationen ermöglichen sowohl die Überprüfung von Handlungskompetenz als auch kommunikativer Fertigkeiten.

## References

[1] Bundesministerium für Gesundheit (2002). Approbationsordnung für Ärzte vom 27. Juni 2002 (BGBl. I S. 2405), die zuletzt durch Artikel 2 der Verordnung vom 22. September 2021 (BGBl. I S. 4335) geändert worden ist.

[2] Bundesministerium für Gesundheit (2020). Verordnung zur Abweichung von der Approbationsordnung für Ärzte bei einer epidemischen Lage von nationaler Tragweite vom 30. März 2020.

[3] Cleland JA, Abe K, Rethans J-J (2009). The use of simulated patients in medical education: AMEE Guide No 42. Med Teach.

[4] Fritsche V, Siol AF, Schnabel KP (2020). Use of simulation patients in the third section of the medical examination. GMS J Med Educ.

[5] Medizinischer Fakultätentag der Bundesrepublik Deutschland e. V. (2021) Nationaler Kompetenzbasierter Lernzielkatalog Medizin – Version 2.0. https://nklm.de/zend/menu. Zugegriffen: 30. Okt. 2022

[6] Overly FL, Sudikoff SN, Duffy S (2009). Three scenarios to teach difficult discussions in pediatric emergency medicine: sudden infant death, child abuse with domestic violence, and medication error. Simul Healthc.

[7] Sommer M, Fritz AH, Thrien C (2019). Simulated patients in medical education—a survey on the current status in Germany, Austria and Switzerland. GMS J Med Educ.

